# Naïve-memory regulatory T cells ratio is a prognostic biomarker for patients with acute ischemic stroke

**DOI:** 10.3389/fnagi.2023.1072980

**Published:** 2023-02-23

**Authors:** Gang Deng, Yue Tang, Jun Xiao, Xin Chen, Yun-Hui Chu, Ke Shang, Luo-Qi Zhou, Chuan Qin, Feng Wang, Dai-Shi Tian

**Affiliations:** ^1^Department of Neurology, Tongji Hospital, Tongji Medical College, Huazhong University of Science and Technology, Wuhan, China; ^2^Department of Laboratory Medicine, Tongji Hospital, Tongji Medical College, Huazhong University of Science and Technology, Wuhan, China

**Keywords:** acute ischemic stroke, prognosis, regulatory T cells, naïve regulatory T cells, memory regulatory T cells

## Abstract

**Background:**

Regulatory T cells (Treg) have been identified as a key modulator of neuroinflammation in stroke. However, little is known about the association of Treg subpopulations with clinical outcome in patients with acute ischemic stroke (AIS).

**Methods:**

Patients within 1 week from stroke onset were prospectively enrolled in this study. Healthy controls were sex-and age-matched 1:1 to AIS patients. The frequencies of Treg and Treg subsets were analyzed by flow cytometry and compared with nonstroke control. Univariate and multivariate logistic regression analysis was performed to investigate the prognostic value of Treg subsets in stroke outcomes.

**Results:**

A total of 328 patients and 328 controls were included in the study. Compared with controls, patients with AIS had higher levels of Treg frequency and memory Treg (mTreg) frequency, but lower levels of naïve Treg (nTreg) frequency and nTreg/mTreg ratio. One hundred twenty-six (38.4%) patients experienced unfavorable outcome (modified Rankin score 2–6). Multivariate regression analysis showed that nTreg/mTreg ratio was negatively associated with unfavorable 90-day outcome (the highest tertile versus the lowest tertile: odds ratio 0.13, 95% confidential interval [CI] 0.05–0.35). The risk estimation of unfavorable 90 day outcome can be significantly improved by adding nTreg/mTreg ratio to the conventional clinical parameters (continuous net reclassification improvement 91.26, 95% CI 69.04–113.5%, *p* < 0.001; integrated discrimination improvement 22.38, 95% CI 17.16–27.59%, *p* < 0.001).

**Conclusion:**

This study showed that patients with AIS had elevated Treg frequency and mTreg frequency, but reduced nTreg frequency and nTreg/mTreg ratio. Admission nTreg/mTreg ratio was an independent predictor of unfavorable 90 day outcome in AIS. However, large sample-size cohort studies are needed to confirm our findings.

## 1. Introduction

Acute ischemic stroke (AIS) is a leading cause of death and disability worldwide. Stroke-triggered neuroinflammation is crucial in the pathogenesis of brain injury and recovery (Jayaraj et al., 2019). Infiltration of various immune cells following stroke is a key component of neuroinflammation in mediating secondary brain injury (Iadecola and Anrather, 2011; Jayaraj et al., 2019). On the other hand, immunological responses after stroke are tightly controlled by regulatory mechanisms to limit secondary tissue damage (Nathan, 2002). Regulatory T cells (Treg), a subset of T lymphocytes, have been shown to restrain neuroinflammation and maintain immune homeostasis, and thus serve as neuroprotective cells (Liesz et al., 2015). However, preclinical studies investigating the effects of Treg on stroke outcome yielded conflicting results. Liesz et al. (2009, 2013) and Xie et al. (2014) found that depletion of Treg in animal models resulted in the increase of infarct size, but others revealed no alteration (Ren et al., 2011; Stubbe et al., 2013) or even reduction of infarct volume (Kleinschnitz et al., 2013). Moreover, there were also inconsistencies in clinical studies investigating circulating Treg numbers and function. While Yan et al. (2012) and Jiang et al., 2017 found that circulating frequencies of Treg were increased following stroke, Ruhnau et al. (2016) and Li et al. (2021) reported a reduced Treg percentage. The discrepancies have been attributed to different infarct sizes or stroke severity, different stroke models or populations (Liesz et al., 2015), as well as Treg being a heterogeneous cell population (Ruhnau et al., 2016).

However, Treg have just been considered as a single cell population in most previous studies. In fact, human Treg can be divided into several subgroups that differ in phenotype and function (Miyara and Sakaguchi, 2011). Human Treg include the CD45RO+ naïve Treg (nTreg) and CD45RA+ memory Treg (mTreg) (Seddiki et al., 2006). Most circulating Treg (90–95%) are mTreg, and their levels are maintained throughout life (Booth et al., 2010). The frequency of nTreg declines with age, mainly due to thymic involution (Seddiki et al., 2006). nTreg expand readily *in vitro*, generating a homogeneous Treg population (Booth et al., 2010). nTreg is the main subpopulation with suppressive activity while mTreg have limited suppressive activity, but can produce pro-inflammatory cytokines (Yang et al., 2017). Decreased nTreg and increased mTreg has been observed in several conditions, such as chronic obstructive pulmonary disease (Yang et al., 2017), non-ST elevation acute coronary syndrome (Zhang et al., 2012), chronic heart failure (Tang et al., 2011), children primary hypertension (Gackowska et al., 2019), Alzheimer’s disease and multiple sclerosis (Ciccocioppo et al., 2019). Reduced nTreg/mTreg ratio was significantly correlated with left ventricular hypertrophy (Gackowska et al., 2018) and blood pressure values in children with primary hypertension (Gackowska et al., 2019). Although Treg has been suggested to be an independent predictor of stroke outcome, the role of its subpopulations in AIS has rarely been investigated. Therefore, we aimed to investigate the prognostic value of the subpopulations of Treg (nTreg, mTreg) and nTreg/mTreg ratio in stroke outcome.

Recent studies have demonstrated that the expression of CD25 in combination with low to negative expression of CD127 can be used as an excellent biomarker of human Treg (Liu et al., 2006). In the current study, we explored the alteration of CD4 + CD25 + CD127 low Treg, nTreg (CD4 + CD25 + CD127 low CD45RA+) and mTreg (CD4 + CD25 + CD127 low CD45RO+) in AIS and investigated their role in stroke outcome prediction.

## 2. Methods

### 2.1. Subjects

We performed a retrospective analysis of a prospectively collected database of consecutive stroke patients at a single stroke center from March 2019 to December 2020 (NCT03122002). Patients with AIS onset within 1 week and with Treg subpopulation data available who was treated medically were included in analysis. Clinical diagnosis of ischemic stroke was confirmed by CT or MR imaging. Each patient was sex-and age-matched with a non-stroke healthy control (the same sex as the matched patient and a difference of age within ±2 years old). Participants with known autoimmune or inflammatory diseases, clinical signs of acute infection on admission, and that who underwent endovascular treatment were excluded. Blood was obtained from participants between 5:00 a.m. and 6:00 a.m. the next morning after they were hospitalized. This study was approved by the Ethics Committee of Tongji Hospital, and written informed consent was obtained from patients or their surrogates before enrollment.

### 2.2. Data collection

Baseline demographic and clinical data, including age, sex, medical history, blood pressure, serum glucose, and treatment with intravenous thrombolysis were all prospectively collected. The stroke severity was evaluated with the NIHSS score (range from 0 to 42) score at their admission by a stroke neurologist. In this study, ischemic stroke was classified into 3 subtypes according to the TOAST criteria: large artery atherosclerosis (LAA), cardiogenic embolism (CE), and other causes.

### 2.3. Flow cytometry

**Figure 1:**
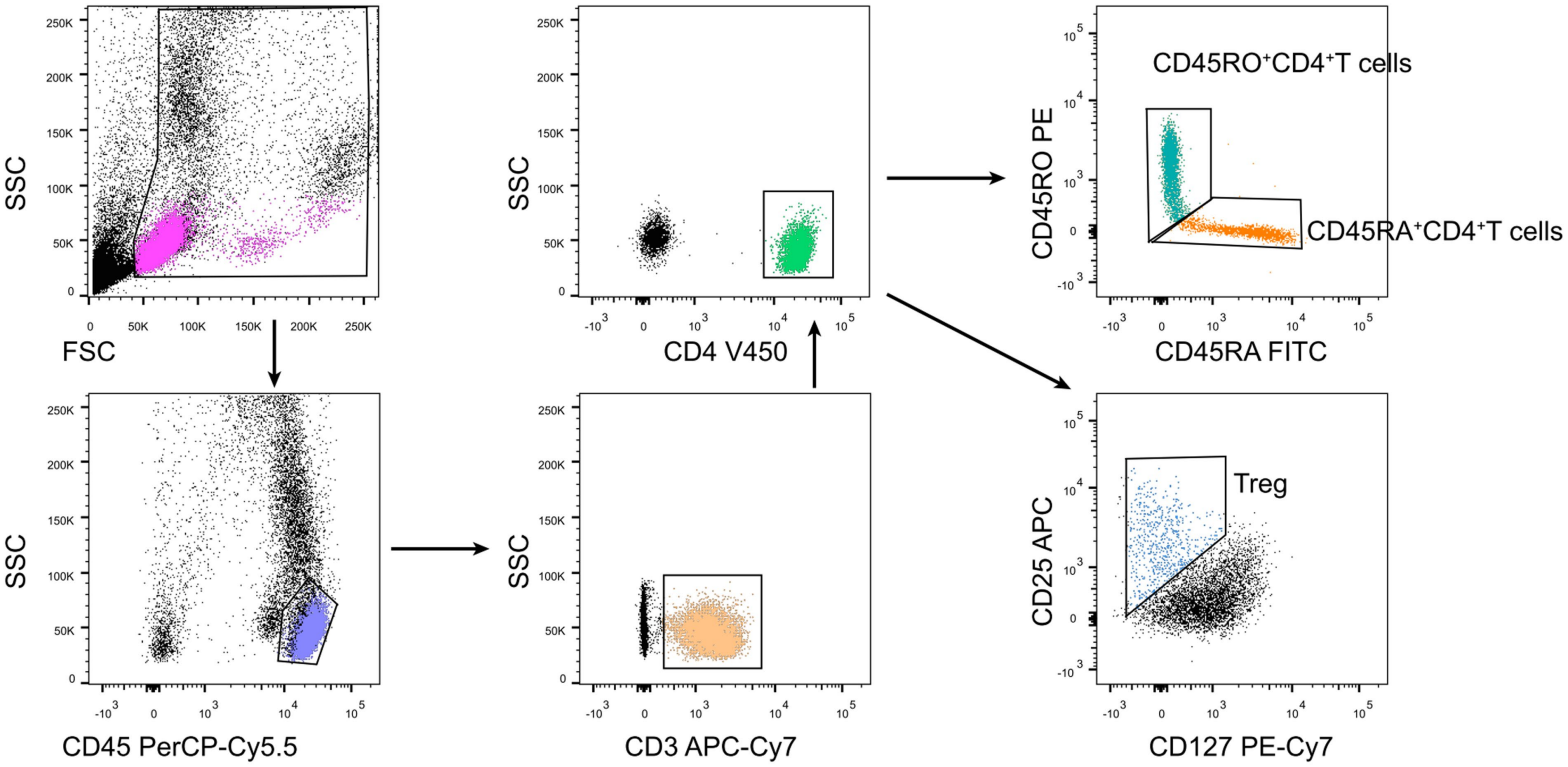
The gating stratege for Treg phenotype analysis.

Heparinized peripheral blood was collected for lymphocyte phenotyping. One hundred μL of whole blood were added with the following monoclonal antibodies: anti-CD3, anti-CD4, anti-CD25, anti-CD127, anti-CD45RA, and anti-CD45RO (BD Biosciences, San Jose, CA, United States). Isotype controls with irrelevant specificities were used as negative controls. Cell suspensions were incubated for 20 min at room temperature. After lysing red blood cells, the cells were washed and resuspended in phosphate buffer saline. Then, the cells were analyzed with FACSCanto flow cytometer. The gating strategies for Treg phenotype analysis were as follows: CD4+ T cells (CD3 + CD4+), Treg (CD3 + CD4 + CD25 + CD127low), nTreg (CD3 + CD4 + CD25 + CD127lowCD45RA+), and mTreg (CD3 + CD4 + CD25 + CD127lowCD45RO+) (Figure 1). The frequencies of Treg, nTreg, and mTreg were calculated by the count of each population divided by the count of CD4+ T cells.

### 2.4. Statistical analysis

Categorical variables were represented as percentage and frequency. Continuous variables were expressed as mean ± SD if distributed normally and otherwise, as median and interquartile range (IQR). For variables with normal distribution, differences between groups were compared using Student’s t-test. Chi-square test and Mann–Whitney *U* rank sum test were used for categorical variables and continuous variables with nonnormal distribution, respectively. Univariate and multivariate binary logistic regressions were performed to assess the associations of admission Treg and differential Treg subsets (both as ordinal variables by tertiles and continuous variables) with 90 day outcomes (mRS 0–1 defined as favorable outcome, mRS 2–6 as unfavorable outcome). Variables with *p* < 0.1 on univariate analyzes, as well as age and NIHSS score, were included in the next multivariate analyzes. Tests for linear trend in risk across Treg subsets tertiles were performed. Subgroup analyzes were also performed according to age, NIHSS score and TOAST classification. Optimal cut-off points were calculated utilizing receiver operating characteristic curve analysis.

Moreover, we calculated C statistics, net reclassification improvement (NRI) and integrated discrimination improvement (IDI) to investigate the added prognostic values of Treg subsets. Calibration plot and its slope was used to evaluate the calibration of the new model. Statistical analysis was conducted using SPSS Statistics version 24.0 (IBM Corp., Armonk, NY, United States) and R Studio 4.2.1 (R Foundation for Statistical Computing, Vienna, Austria). A two-tailed value of *p* < 0.05 was considered significant.

## 3. Results

A total of 328 patients with AIS and matched 328 controls were included in the analysis. The mean ages were 55.9 ± 11.0 and 55.7 ± 11.5 years, respectively. The male proportion was 80.8% for each group. The two groups were comparable in age, sex, proportions of hypertension, diabetes mellitus, dyslipidemia, coronary heart disease, atrial fibrillation, and current smokers, alcohol consumption, and levels of SBP, DBP and serum glucose. For the AIS group, the median NIHSS score on admission was 3 points (IQR, 1–5); and 236 (72.0%) and 10 (3.0%) participants had large artery atherosclerotic and cardioembolic ischemic strokes, respectively. The main baseline characteristics of the 2 groups are shown in Table 1.

**Table 1:** Baseline characteristics of controls and participants with acute ischemic stroke (AIS).

	Control group (*n* = 328)	AIS group (*n* = 328)	*P*
Age, yrs., median (IQR)	55.9 ± 11.0	55.7 ± 11.5	0.868
Sex, male, N (%)	265 (80.8)	265 (80.8)	1.000
Hypertension, N (%)	141 (43.0)	148 (45.1)	0.582
DM, N (%)	59 (18.0)	67 (20.4)	0.428
Dyslipidemia, N (%)	68 (20.7)	80 (24.4)	0.262
Coronary heart disease, N (%)	25 (7.6)	26 (7.9)	0.884
Atrial fibrillation, N (%)	16 (4.9)	15 (4.6)	0.854
Current smokers, N (%)	131 (39.9)	136 (41.5)	0.691
Alcohol consumption, N (%)	124 (37.8)	132 (40.2)	0.522
SBP, mmHg, median (IQR)	130 (126–151)	136 (127–155)	0.242
DBP, mmHg, median (IQR)	82 (75–90)	83 (77–93)	0.224
Serum glucose, mmol/L, median (IQR)	5.33 (4.84–6.39)	5.37 (4.94–6.42)	0.301
NIHSS, median (IQR)	–	3 (1–5)	–
TOAST			
LAA	–	236 (72.0)	–
CE	–	10 (3.0)	
Other	–	82 (25.0)	
Tregs frequency, median (IQR)	3.28(3.85–4.85)	3.85 (4.59–5.20)	<0.001
nTregs frequency, median (IQR)	0.67 (0.94–1.38)	0.51 (0.83–1.23)	<0.001
mTregs frequency, median (IQR)	2.52 (3.08–3.68)	3.02 (3.51–4.16)	<0.001
nTreg/mTregs ratio, median (IQR)	0.31 (0.24–0.43)	025 (0.18–0.36)	<0.001

IQR, interquartile range; DM, diabetes mellitus; SBP, systolic blood pressure; DBP, diastolic blood pressure; NIHSS, National Institute of Health Stroke Scale; TOAST, trial of org 10,172 in acute stroke treatment; LAA, large artery atherosclerosis; CE, cardiogenic embolism; Treg, regulatory T cells; nTreg, naïve regulatory T cells; mTreg, memory regulatory T cells.

Compared with the control group, the AIS group had higher levels of Treg frequency (median, 3.28% versus 3.85%; IQR, 3.85–4.85% versus 4.59–5.20%; *p* < 0.001) and mTreg frequency (median 2.52% versus 3.02%; IQR 3.08–3.68% versus 3.51–4.16%; *p* < 0.001), but lower levels of nTreg frequency (median 0.67% versus 0.51%; IQR 0.94–1.38% versus 0.83–1.23%; *p* < 0.001) and nTreg/mTreg ratio (median 0.31 versus 0.25; IQR 0.24–0.43 versus 0.18–0.36; *p* < 0.001) (Table 1).

Of the 328 patients with AIS, 126 (38.4%) had an unfavorable outcome. The main baseline characteristics according to 90 day neurological outcome are shown in Table 2. Patients with unfavorable outcome had higher levels of SBP and serum glucose, more severe stroke (defined as NIHSS score), and more proportion of LAA-related stroke.

**Table 2:** Baseline characteristics of patients with acute ischemic stroke according to 90-day neurological outcome (modified Rankin Scale 0–1 versus 2–6).

Variables	Favorable outcome group (*n* = 202)	Unfavorable outcome group (*n* = 126)	*P*
Age, yrs., median (IQR)	55.0 (50.0–63.0)	57.0 (50.0–62.0)	0.229
Sex, male, N (%)	168 (83.2)	102 (81.0)	0.609
Hypertension, N (%)	114(56.4)	80 (63.5)	0.206
DM, N (%)	52 (25.7)	32 (25.4)	0.944
Dyslipidemia, N (%)	54 (26.7)	26 (20.6)	0.211
Coronary heart disease, N (%)	16 (7.9)	10 (7.9)	0.996
Atrial fibrillation, N (%)	6 (3.0)	4 (3.2)	0.917
History of stroke, N (%)	36 (17.8)	16 (12.7)	0.217
Current smokers, N (%)	88 (43.6)	48 (38.1)	0.328
Alcohol consumption, N (%)	82 (40.6)	64 (50.8)	0.071
SBP, mmHg, median (IQR)	135 (123–152)	148 (131–159)	<0.001
DBP, mmHg, median (IQR)	83 (77–95)	84 (77–91)	0.878
Serum glucose, mmol/L, median (IQR)	5.23 (4.84–6.22)	5.78 (5.03–6.50)	0.003
NIHSS, median (IQR)	1 (0–2)	5 (4–8)	<0.001
TOAST, N (%)			<0.001
LAA	132 (65.3)	98 (77.8)	
CE	22 (10.9)	20 (15.9)	
Other	48 (23.8)	8 (6.3)	
Treatment with iv-rtPA, N (%)	26 (12.9)	14 (11.1)	0.636
Tregs frequency, median (IQR)	4.56 (3.83–5.29)	4.71 (3.86–5.28)	0.595
Tregs frequency, N (%)			0.089
Tertile 1	68 (33.7)	38 (30.2)	
Tertile 2	60 (29.7)	52 (41.3)	
Tertile 3	74 (36.6)	36 (28.6)	
nTregs frequency, median (IQR)	0.98 (0.75–1.38)	0.82 (0.55–1.23)	0.011
nTregs frequency, N (%)			0.002
Tertile 1	54 (26.7)	54 (42.9)	
Tertile 2	72 (35.6)	38 (30.2)	
Tertile 3	76 (37.6)	34 (27.0)	
mTregs frequency, median (IQR)	3.57 (2.92–4.08)	3.22 (3.55–4.34)	0.264
mTregs frequency, N (%)			0.212
Tertile 1	72 (35.6)	34 (27.0)	
Tertile 2	62 (30.7)	48 (38.1)	
Tertile 3	68 (33.7)	44 (34.9)	
nTreg/mTreg ratio, median (IQR)	0.35 (0.26–0.52)	0.15 (0.20–2.26)	<0.001
nTreg/mTreg ratio, N (%)			0.001
Tertile 1	52 (25.7)	57 (45.2)	
Tertile 2	65 (32.2)	36 (28.6)	
Tertile 3	85 (42.1)	33 (26.2)	

IQR, interquartile range; DM, diabetes mellitus; SBP, systolic blood pressure; DBP, diastolic blood pressure; NIHSS, National Institute of Health Stroke Scale; TOAST, trial of org 10,172 in acute stroke treatment; LAA, large artery atherosclerosis; CE, cardiogenic embolism; Treg, regulatory T cells; nTreg, naïve regulatory T cells; mTreg, memory regulatory T cells.

In our cohort, there were no significant differences in Treg and mTreg frequency between patients with favorable and unfavorable outcomes. However, patients with unfavorable outcome had lower level of nTreg frequency (median 0.82% versus 0.98%; IQR 0.55–1.23% versus 0.75–1.38%; *p* = 0.011) and lower nTreg/mTreg ratio (median 0.15 versus 0.35; IQR 0.20–0.26 versus 0.26–0.52; *p* < 0.001) (Table 2).

In univariate analysis, nTreg frequency and nTreg/mTreg ratio were significantly associated with unfavorable outcome (*P* for trend = 0.01 and 0.001, respectively) while Treg and mTreg frequency were not (*P* for trend = 0.091 and 0.214, respectively) (Table 3). nTreg frequency, mTreg frequency and nTreg/mTreg ratio were subjected for further multivariate analysis as their *P* for trend were all less than 0.1.

**Table 3:** Univariate and multivariate logistic regression analyzes depicting the associations of admission Treg subpopulation with 90 day unfavorable outcome.

Treg subpopulation	Unadjusted OR	*P*	*P* for trend	Adjusted OR^a^	*P*	*P* for trend
Treg			0.091			0.117
Tertile 1	Reference			Reference		
Tertile 2	1.55 (0.90–2.67)	0.114		2.31 (0.89–5.98)	0.086	
Tertile 3	0.87 (0.50–1.53)	0.629		0.88 (0.36–2.16)	0.784	
nTreg			0.01			0.057
Tertile 1	Reference			Reference		
Tertile 2	0.53 (0.31–0.91)	0.021		1.10 (0.45–2.73)	0.832	
Tertile 3	0.45 (0.26–0.78)	0.004		0.36 (0.14–0.95)	0.039	
mTreg			0.214			-
Tertile 1	Reference			-		
Tertile 2	1.64 (0.94–2.86)	0.081		-	-	
Tertile 3	1.37 (0.79–2.39)	0.268		-	-	
nTreg-mTreg ratio			0.001			<0.001
Tertile 1	Reference			Reference		
Tertile 2	0.51 (0.29–0.90)	0.019		0.23 (0.08–0.65)	0.005	
Tertile 3	0.35 (1.63–5.09)	<0.001		0.13 (0.05–0.35)	<0.001	

a Adjusted for age, systolic blood pressure, NIHSS score, and TOAST classification.

OR, odds ratio; Treg, regulatory T cells; nTreg, naïve regulatory T cells; mTreg, memory regulatory T cells; NIHSS, National Institute of Health Stroke Scale.

**Figure 2:**
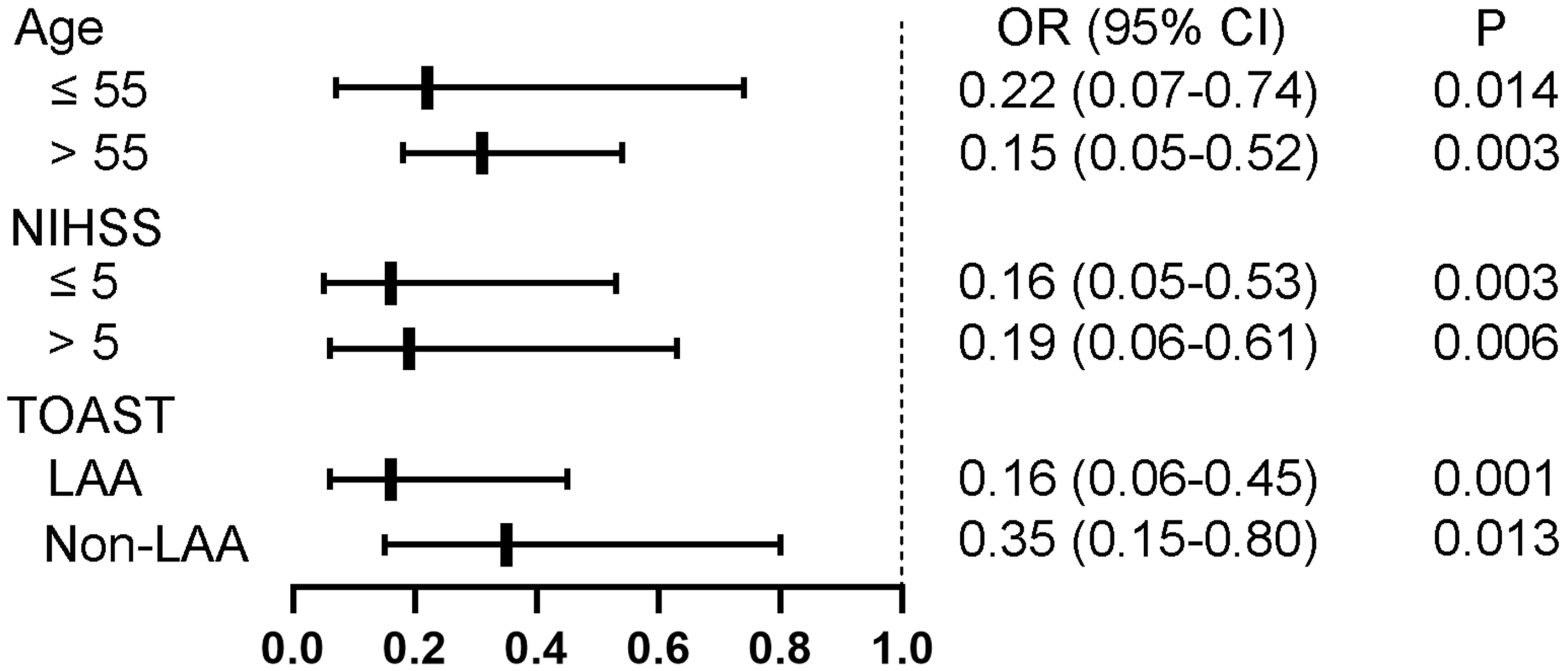
Subgroup analysis regarding nTreg/mTreg ratio according to age, NIHSS score, and TOAST classification. nTreg, naïve regulatory T cells; mTreg, memory regulatory T cells. NIHSS, National Institute of Health Stroke Scale; TOAST, trial of org 10,172 in acute stroke treatment; LAA, large artery atherosclerosis.

To minimize the influence of collinearity between these three parameters on the regression analysis, separate models were run for them. After adjustment for age, SBP, NIHSS score and TOAST classification, only nTreg/mTreg ratio remained significantly associated with unfavorable 90 day outcome (Table 3). Compared with the patients in the lowest tertile of nTreg/mTreg ratio, the adjusted ORs for the middle and highest tertile were 0.23 (95% CI: 0.08–0.65) and 0.13 (95% CI: 0.05–0.35), respectively. Each 0.1 point increase of the nTreg/mTreg ratio was also negatively associated with unfavorable 90 day outcome (OR 0.67, 95% CI 0.51–0.87). Moreover, subgroup analyzes showed that the association between nTreg/mTreg ratio and unfavorable 90-day outcome remained significant according to age (≤55 versus > 55 years old), NIHSS score (≤5versus > 5 points) and TOAST classification (LAA versus. non-LAA-related stroke) (Figure 2). At an nTreg/mTreg ratio cut-off point of 0.30, the sensitivity and specificity for favorable 90 day outcome were 79.0 and 76.0%, respectively (*C* statistic: 0.79, 95% CI: 0.73–0.85, *p* < 0.001).

**Figure 3:**
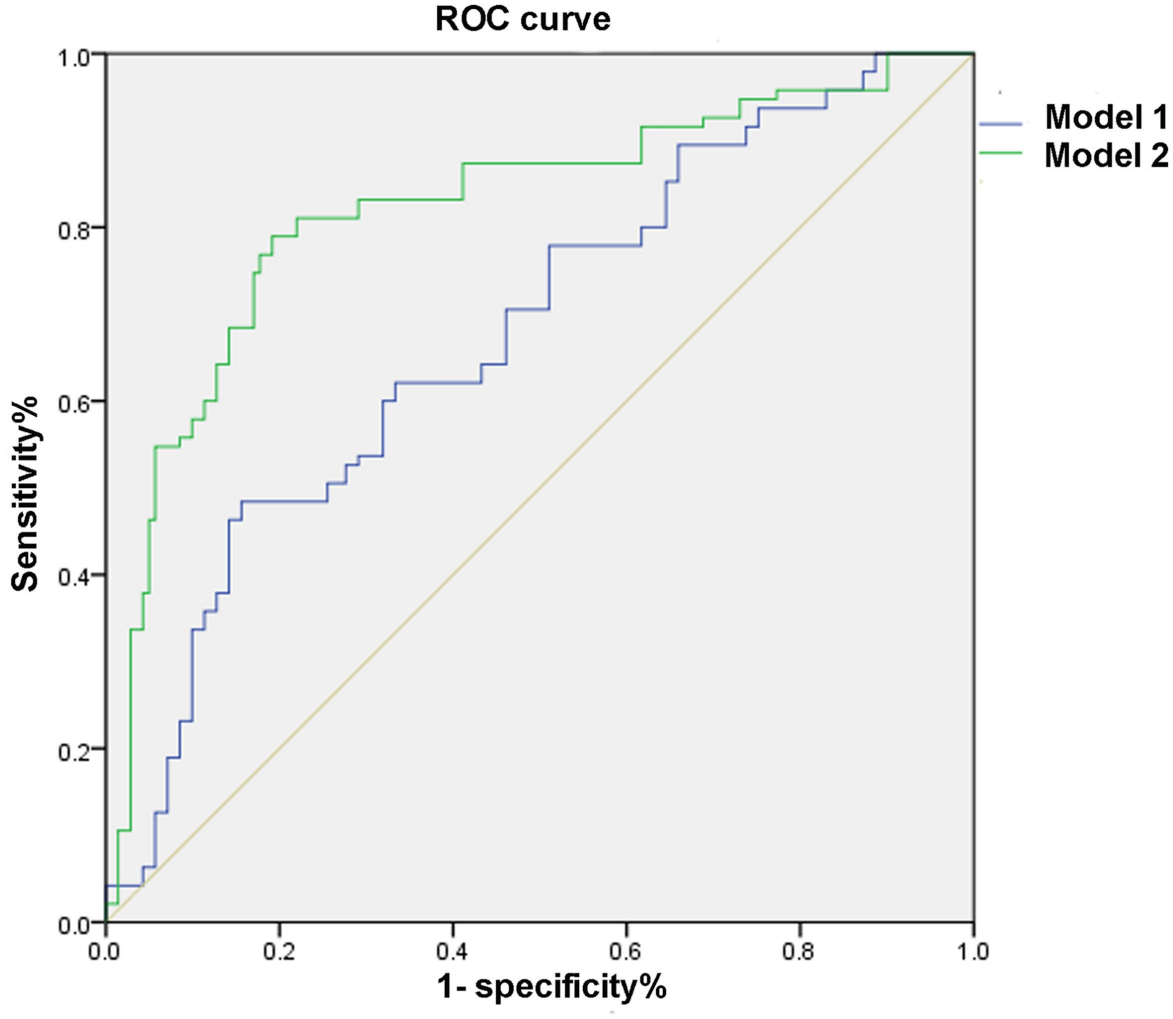
Receiver operating characteristic curve. Model 1: adjusted for age, systolic blood pressure, NIHSS score, and TOAST classification. Model 2: Model 1 plus nTreg/mTreg ratio. ROC, receiver operating characteristic curve; nTreg, naïve regulatory T cells; mTreg, memory regulatory T cells.

**Figure 4:**
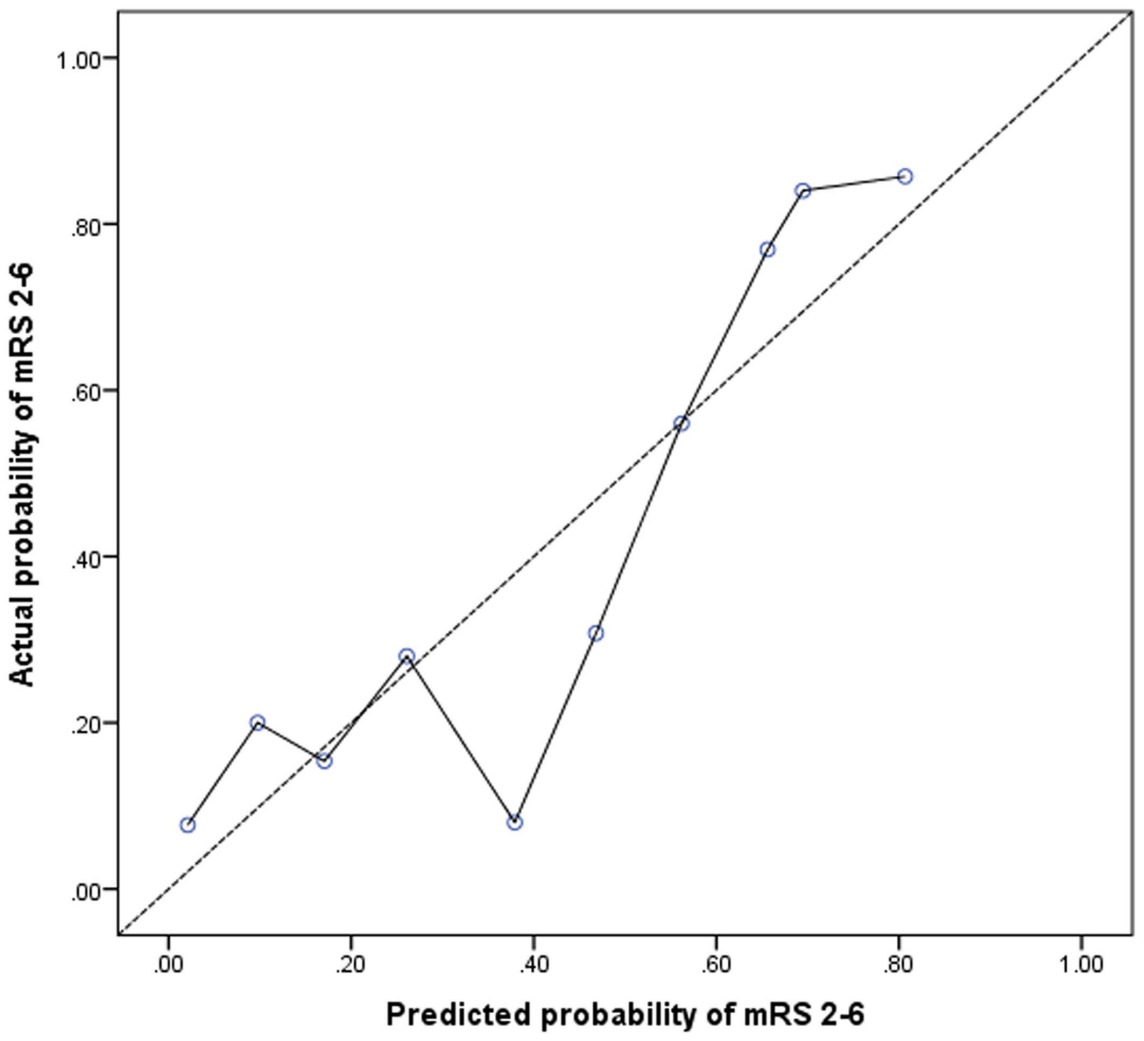
Calibration plot for the model containing age, systolic blood pressure, NIHSS score, TOAST classification, and nTreg/mTreg ratio. A calibration slope of 1.0 indicates perfect calibration (dotted line). nTreg, naïve regulatory T cells; mTreg, memory regulatory T cells.

Adding nTreg/mTreg ratio to a prediction model containing age, SBP, admission NIHSS score and TOAST classification significantly improved its predictive ability (C-statistic 0.69 versus 0.82, 95%CI 0.62–0.76 versus 0.77–0.88, *p* = 0.003) (Table 4 and Figure 3). The continuous NRI was 91.26% (95% CI: 69.04–113.5%, *p* < 0.001). IDI was 22.38% (95% CI: 17.16–27.59%, *p* < 0.001) (Table 4). The calibration plot is shown in Figure 4. The slope of the plot was 0.82, indicating that the model was reasonably calibrated.

**Table 4:** Discrimination and reclassification statistics for unfavorable 90-day outcome by circulatory nTreg/mTreg ratio.

Model	C statistic		NRI (continuous), %		IDI, %	
	Estimate (95% CI)	*P*	Estimate (95% CI)	*P*	Estimate (95% CI)	*P*
Model 1^a^	0.69 (0.62–0.76)		Reference		Reference	
Model 2^b^	0.82 (0.77–0.88)	0.003	0.91 (0.69–1.13)	<0.001	0.22 (0.17–0.28)	<0.001

a Model 1 adjusted for age, systolic blood pressure, TOAST classification and admission NIHSS score.

b Model 2 adjusted for model 1 plus admission circulatory nTreg/mTreg ratio.

NRI, net reclassification improvement; IDI, integrated discrimination improvement; CI, confidence interval; NIHSS, National Institutes of Health Stroke Scale; nTreg, naïve regulatory T cells; mTreg, memory regulatory T cells.

## 4. Discussion

In this study, we observed an elevation of circulating Treg and mTreg frequencies, in parallel with the reduction of nTreg frequency and nTreg/mTreg ratio in patients with AIS. Moreover, our results suggest that peripheral nTreg/mTreg ratio was an independent predictor of unfavorable stroke outcome.

Treg play a crucial part in the modulation of immune responses and the control of detrimental immune activation due to their immunoregulatory and immunosuppressive functions (Sakaguchi et al., 2006). Most preclinical and clinical studies suggest that Treg serve as protective components after stroke (Liesz et al., 2015). In line with previous studies, we found that Treg frequency was increased in stroke patients (Yan et al., 2012; Jiang et al., 2017). Nevertheless, contrary to a previous study (Li et al., 2021), we failed to observe a prognostic value of Treg in stroke outcome. The difference between the studies of ours and others could result from different patient characteristics including comorbidities and stroke severity, as well as Treg being a heterogeneous cell population.

However, the distribution of Treg and their role in stroke has rarely been investigated. Treg (CD4 + CD25 + CD127low) can be further divided into naïve Treg (nTreg, CD4 + CD25 + CD127lowCD45RA+) and memory Treg (mTreg, CD4 + CD25 + CD127lowCD45RO+) according to the expression of CD45RA and CD45RO (Seddiki et al., 2006). Most of Treg in the peripheral blood of human adults are CD45RO+ mTreg, which, contrary to CD45RA+ nTreg, do not express the lymph node homing receptors CCR7 or CD62L, nor FOXP3, the master regulator of Treg. In addition, the subset of mTreg also includes cells secreting interleukin-2 (IL-2), interferon-γ (IFN-γ) and IL-10 and shows less robust suppressive activity after *in vitro* expansion compared to nTreg (Hoffmann et al., 2006). The observed alteration of Treg subpopulations (decreased nTreg and increased mTreg) in our cohort of AIS patients was contrary to a previous report (Ruhnau et al., 2016), which showed unchanged nTreg and loss of activated Treg (CD4 + FoxP3 + CD39+), but had a relatively small sample size (48 patients and 26 controls) and unmatched baseline characteristics.

The imbalance of Treg subpopulations (decreased nTreg and increased mTreg) has been observed in chronic obstructive pulmonary disease (Yang et al., 2017), non-ST elevation acute coronary syndrome (Zhang et al., 2012), Alzheimer’s disease and multiple sclerosis (Ciccocioppo et al., 2019). However, the exact role of this Treg redistribution in disease pathogenesis remains elusive. The observed imbalance of nTreg and mTreg in our study may also influence immune regulation mechanisms in the acute stage of AIS.

In this study, we found that circulating nTreg/mTreg ratio was negatively associated with unfavorable outcome in AIS, which means impaired immunomodulatory activity at the early stage of AIS may contribute to unfavorable outcome. To the best of our knowledge, this is the first report evaluating the prognostic value of Treg subsets in stroke outcome. As mentioned above, nTreg is the main subpopulation with suppressive activity while mTreg have limited suppressive activity, but can produce pro-inflammatory cytokines (Yang et al., 2017). The reduced nTreg/mTreg ratio implis a disequilibrium between anti-inflammatory and pro-inflammatory subpopulation which may induce prolonged immune activation and be detrimental for stroke recovery. The prognostic value of nTreg/mTreg ratio was to some extent robust because it was based on a prospectively collected database of consecutive stroke patients with relatively larger sample size and blinded outcome assessment. We also collected and compared related confounding factors and conducted multivariate adjustments. Moreover, the association of nTreg/mTreg ratio with unfavorable 90-day outcome remained significant either analyzed as both a continuous variable and an ordinal variable by tertiles. At last, subgroup analysis according to age, NIHSS score and TOAST classification did not reveal any variation to the association. Therefore, this study allowed a valid assessment of the prognostic value of nTreg/mTreg ratio in AIS.

There are several limitations in our study. Firstly, the sample size may be not sufficient to perform more comprehensively adjustment for other confounders, such as laboratory and imaging parameters. Secondly, Treg can be further subdivided into other biomarkers functionally (i.e., CD31), which allows a more precise evaluation of the role of Treg in stroke. But this did not influence our clinical observation, though the simple characterization of Treg subsets limit the exploration of related pathophysiological mechanisms of stroke. Thirdly, the temporal change of Treg subsets in AIS was not observed. Finally, the symptoms of the included participants were generally mild–moderate stroke with low NIHSS score, so the applicability of our results to the whole population with AIS should be questioned. Thus, the prognostic role of nTreg/mTreg ratio in patients with more severe stroke needs further verification in larger prospective trials.

In conclusion, we found nTreg/mTreg ratio was an independent predictor of unfavorable 90 day outcome in patients with mild–moderate AIS. Larger sample and multicenter studies are needed to confirm our results, and interventions targeting nTreg/mTreg ratio may provide a potential treatment strategy for stroke in the future.

## Data availability statement

The original contributions presented in the study are included in the article/Supplementary material, further inquiries can be directed to the corresponding authors.

## Ethics statement

The studies involving human participants were reviewed and approved by the Ethics Committee of Tongji Hospital, Tongji Medical College, Huazhong University of Science and Technology. The patients/participants provided their written informed consent to participate in this study.

## Author contributions

GD and YT designed and conceived the study and wrote the first draft of the manuscript. JX, XC, Y-HC, KS, and L-QZ collected clinical and radiological data and performed laboratory analysis. GD, YT, and JX conducted the statistical analysis. CQ, FW, and D-ST supervised the study and critically revised the manuscript. D-ST obtained funding. All authors contributed to the article and approved the submitted version.

## Funding

This study was supported by the National Natural Science Foundation of China (Nos. 81873743 and 82071380).

## Conflict of interest

The authors declare that the research was conducted in the absence of any commercial or financial relationships that could be construed as a potential conflict of interest.

## Publisher’s note

All claims expressed in this article are solely those of the authors and do not necessarily represent those of their affiliated organizations, or those of the publisher, the editors and the reviewers. Any product that may be evaluated in this article, or claim that may be made by its manufacturer, is not guaranteed or endorsed by the publisher.
